# Molecular Characterization of *bla*_NDM_-Carrying IncX3 Plasmids: *bla*_NDM-16b_ Likely Emerged from a Mutation of *bla*_NDM-5_ on IncX3 Plasmid

**DOI:** 10.1128/spectrum.01449-22

**Published:** 2022-07-13

**Authors:** Tsukasa Ariyoshi, Kotaro Aoki, Hiroaki Kubota, Kenji Sadamasu, Yoshikazu Ishii, Kazuhiro Tateda

**Affiliations:** a Department of Microbiology and Infectious Diseases, Toho University Graduate School of Medicine, Tokyo, Japan; b Department of Microbiology and Infectious Diseases, Toho University School of Medicine, Tokyo, Japan; c Department of Microbiology, Tokyo Metropolitan Institute of Public Health, Tokyo, Japan; Louis Stokes Cleveland VAMC

**Keywords:** carbapenem-resistant *Enterobacterales*, IncX3, plasmid, *bla*_NDM-16b_, whole-genome sequencing, comparative analysis

## Abstract

Dissemination of *bla*_NDM_, which is carried on the IncX3 plasmid, among *Enterobacterales* has been reported worldwide. In particular, *bla*_NDM-5_-carrying IncX3 plasmids can spread among several hosts, facilitating their dissemination. Other variants, such as *bla*_NDM-17_-, *bla*_NDM-19_-, *bla*_NDM-20_-, *bla*_NDM-21_-, and *bla*_NDM-33_-carrying IncX3 plasmids, have also been reported. Here, we characterized, using whole-genome sequencing (WGS), a *bla*_NDM-16b_-carrying IncX3 plasmid harbored by Escherichia coli strain TA8571, which was isolated from a urine specimen of a hospital inpatient in Tokyo, Japan. The *bla*_NDM-16b_ differed in sequence from *bla*_NDM-5_ (C > T at site 698, resulting in an Ala233Val substitution). This *bla*_NDM-16b_-carrying IncX3 plasmid (pTMTA8571-1) is 46,161 bp in length and transferred via conjugation. Transconjugants showed high resistance to β-lactam antimicrobials (except for aztreonam). Because pTMTA8571-1, which carries the Tn*125-*related region containing *bla*_NDM_ and conjugative transfer genes, was similar to the previously reported IncX3 plasmids, we performed phylogenetic analysis based on the sequence of 34 shared genes in 142 *bla*_NDM_-carrying IncX3 plasmids (22,846/46,923 bp). Comparative analysis of the shared genes revealed short branches on the phylogenetic tree (average of 1.08 nucleotide substitutions per shared genes), but each *bla*_NDM_ variant was divided into separate groups, and the structure of the tree correlated with the flowchart of *bla*_NDM_ nucleotide substitutions. The *bla*_NDM_-carrying IncX3 plasmids may thereby have evolved from the same ancestral plasmid with subsequent mutation of the *bla*_NDM_. Therefore, pTMTA8571-1 likely emerged from a *bla*_NDM-5_-carrying IncX3 plasmid. This study suggested that the spread of *bla*_NDM_-carrying IncX3 plasmids may be a hotbed for the emergence of novel variants of *bla*_NDM_.

**IMPORTANCE** *bla*_NDM_-carrying IncX3 plasmids have been reported worldwide. Harbored *bla*_NDM_ variants were mainly *bla*_NDM-5_, but there were also rare variants like *bla*_NDM-17_, *bla*_NDM-19_, *bla*_NDM-20_, *bla*_NDM-21_, and *bla*_NDM-33_, including *bla*_NDM-16b_ detected in this study. For these plasmids, previous reports analyzed whole genomes or parts of sequences among a small number of samples, whereas, in this study, we performed an analysis of 142 *bla*_NDM_-carrying IncX3 plasmids detected around the world. The results showed that regardless of the *bla*_NDM_ variants, *bla*_NDM_-carrying IncX3 plasmids harbored highly similar shared genes. Because these plasmids already spread worldwide may be a hotbed for the emergence of rare or novel variants of *bla*_NDM_, increased attention should be paid to *bla*_NDM_-carrying IncX3 plasmids in the future.

## INTRODUCTION

Carbapenem-resistant *Enterobacterales* (CRE) infections refer to all infections caused by *Enterobacterales* that are resistant to carbapenems and broad-spectrum β-lactams, which are the first-choice antimicrobial agents used to treat infections caused by Gram-negative bacteria (1). CRE infections are divided into carbapenemase-producing *Enterobacterales* (CPE) and non-CPE. Infections caused by CPE are rapidly spreading in health care settings and are associated with a high mortality rate; they are consequently a focus of clinical and public health attention (2). One of the major CPE, New Delhi metallo-β-lactamase (NDM)-producer, was first reported in 2009 after being isolated from a Swedish patient who had been admitted to an Indian hospital (3). Currently, NDM-producing CPEs are spreading rapidly worldwide. In particular, dissemination of the *bla*_NDM-5_ and *bla*_NDM-7_ genes, carried on the IncX3 plasmid, among different species of *Enterobacterales* has been widely reported (4–8). In addition, other *bla*_NDM_ variants carried on IncX3 plasmids, namely, *bla*_NDM-17_, *bla*_NDM-19_, *bla*_NDM-20_, *bla*_NDM-21_, and *bla*_NDM-33_, have been reported (9–13). The role of the IncX3 plasmid in mediating the dissemination of *bla*_NDM_ has therefore been considered (14).

In this study, we report, for the first time, the detection of a *bla*_NDM-16b_-carrying IncX3 plasmid. *bla*_NDM-16b_ was the reassigned name from *bla*_NDM-16_ in 2021 (15). For molecular characterization and to estimate the emergence of this plasmid, we performed whole-genome sequencing analysis, comparative analysis of *bla*_NDM_-carrying IncX3 plasmids that have spread worldwide, antimicrobial susceptibility tests, and bacterial conjugation experiments.

## RESULTS AND DISCUSSION

### Isolate characteristics.

The antimicrobial susceptibility testing results showed that the TA8571 strain was not susceptible to β-lactams, gentamicin, ciprofloxacin, or trimethoprim/sulfamethoxazole, but it was susceptible to amikacin (Table 1). The TA8571 strain tested positive by use of the modified carbapenem inactivation method (mCIM). PCR screening of carbapenemase genes and sequencing resulted in detecting *bla*_NDM-16b_. The *bla*_NDM-16b_ encoding NDM-16b differs from *bla*_NDM-5_ by a cytosine-to-thymine nucleotide substitution at position 698, which causes an amino acid substitution of Ala233Val.

**TABLE 1:** Antimicrobial susceptibility of E. coli strain TA8571 and its transconjugants^*a*^

Antimicrobial agent	MIC (μg/mL) of:		
	E. coli TA8571 (donor)	J53/pTMTA8571-1 (transconjugant)	E. coli J53 (recipient)
Piperacillin	>256 (R)	128 (R)	2 (S)
Piperacillin/tazobactam	256/4 (R)	128/4 (R)	2/4 (S)
Ceftazidime	>256 (R)	>256 (R)	0.25 (S)
Cefotaxime	>256 (R)	>256 (R)	0.25 (S)
Cefepime	64 (R)	32 (R)	0.25 (S)
Moxalactam	>256 (R)	>256 (R)	0.5 (S)
Aztreonam	8 (I)	0.12< (S)	<0.12 (S)
Imipenem	8 (R)	8 (R)	0.25 (S)
Meropenem	16 (R)	16 (R)	<0.12 (S)
Amikacin	8 (S)	4 (S)	4 (S)
Gentamicin	32 (R)	0.5 (S)	0.5 (S)
Ciprofloxacin	128 (R)	<0.12 (S)	<0.12 (S)
Trimethoprim/sulfamethoxazole	>32/608 (R)	0.125/2.4 (S)	0.25/4.8 (S)

a Breakpoints determined using CLSI M100-ED31. R, resistance; I, intermediate resistance; S, susceptibility.

### Whole-genome sequencing of TA8571 strain.

The complete genome sequence of the TA8571 strain with 289× coverage is shown in Table 2. TA8571 strain was identified as Escherichia coli by average nucleotide identity (ANI) analysis (ANI value of 96.98% for E. coli type strain genome). The sequence type (ST) for the chromosome was ST746. The genome of the TA8571 strain comprised a chromosome and 4 plasmids and carried 13 acquired antimicrobial resistance genes and 4 chromosomal point mutations in quinolone resistance-determining regions (Table 2). *bla*_NDM-16b_ was the only one found on a plasmid; the other acquired antimicrobial resistance genes were chromosomally encoded.

**TABLE 2:** Whole-genome information of E. coli TA8571^*a*^

Replicon	Length (bp)	MLST	Inc type	Acquired antimicrobial resistance gene(s)	Chromosomal point mutations in QRDRs (amino acid substitution[s])	GenBank accession no.
Chromosome	4,751,019	ST746	NA	*aadA5*, *aac(3)-IId*, *aph(3′)-lb*, *aph(6)-ld*, *mdf(A)*, *mph(A)*, *erm(B)*, *sul1*, *sul2*, *tet(A)*, *dfrA17*, *bla*_CTX-M-14_	*gyrA* (S83L, D87N), *parE* (L416F), *parC* (S80I)	AP024205
pTMTA8571-1	46,161	NA	IncX3	*bla*_NDM-16b_	NA	AP024206
pTMTA8571-2	15,626	NA	—	—	NA	AP024207
pTMTA8571-3	3,397	NA	—	—	NA	AP024208
pTMTA8571-4	2,444	NA	—	—	NA	AP024209

a NA, not acceptable; —, not found; QRDRs, quinolone resistance-determining regions.

### Characteristics of pTMTA8571-1 and transconjugants.

**FIG 1:**
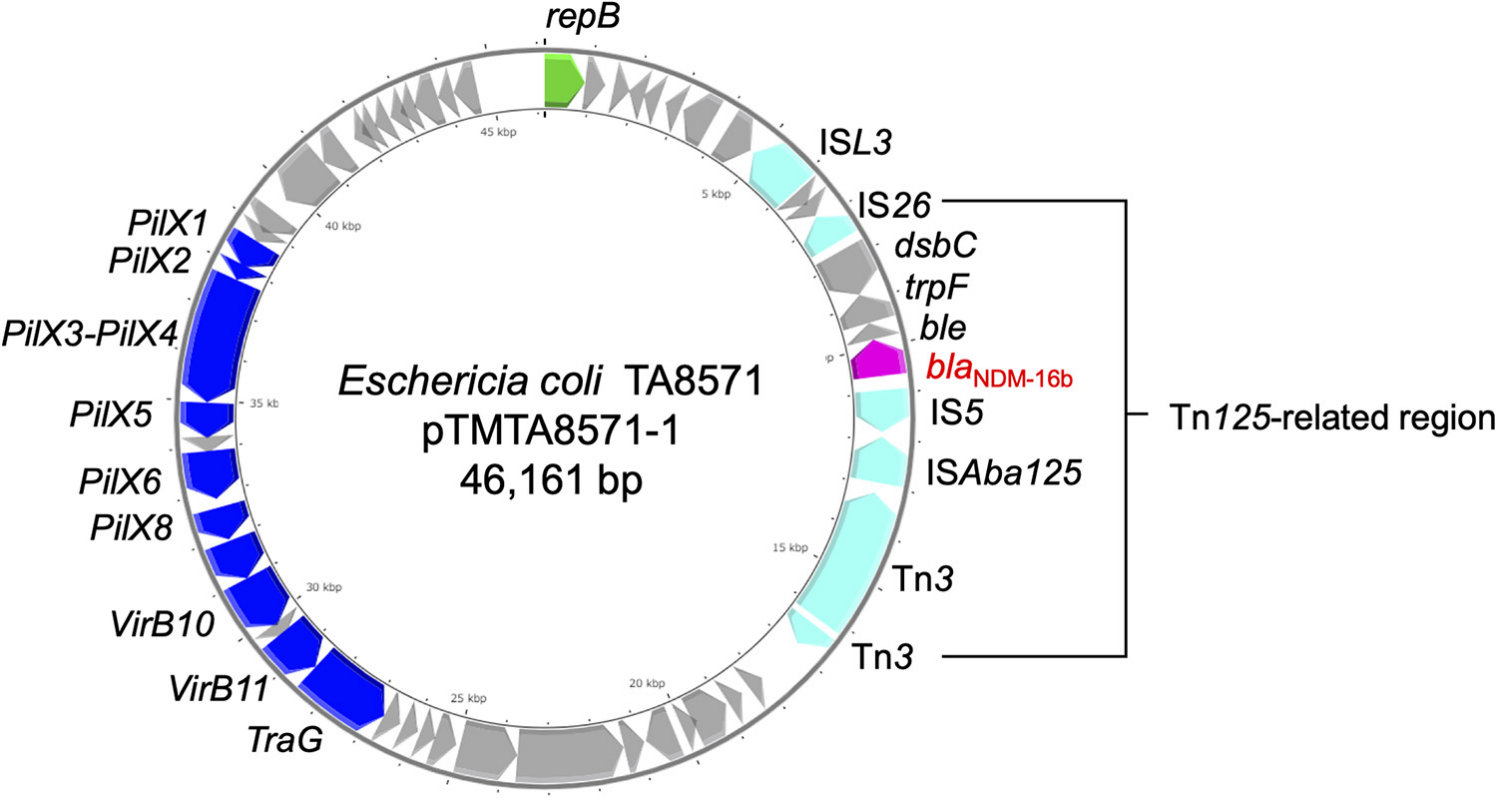
Genome structure of pTMTA8571-1. Arrows indicate predicted open reading frames. The color code is as follows: green, replication initiation protein genes; blue, conjugal transfer genes; cyan, transposase genes; magenta, antibiotic resistance genes. The other genes are represented by gray arrows.

pTMTA8571-1 was found to be a 46,161-bp *bla*_NDM-16b_-carrying IncX3 plasmid in which *bla*_NDM-16b_ was encoded in the Tn*125*-related region (Fig. 1). The Tn*125*-related region comprised genes related to horizontal transfer, such as IS*26*, *trpF*, and IS*Aba125* (Fig. 1). pTMTA8571-1 could be transferred by conjugation to E. coli J53. The conjugal transfer frequency was approximately 2.0 × 10^−5^. According to S1 pulsed-field gel electrophoresis (see Fig. S1 in the supplemental material), the transconjugant contained a plasmid of approximately 50 kbp that was confirmed to carry *bla*_NDM-16b_ by sequencing and lack *bla*_CTX-M-14_ by PCR. The antimicrobial susceptibility testing results showed that transconjugants (J53/pTMTA8571-1) were resistant to β-lactams, except for aztreonam, and were susceptible to all other antimicrobials tested (Table 1).

### Comparative analysis of *bla*_NDM_-carrying IncX3 plasmids.

**FIG 2:**
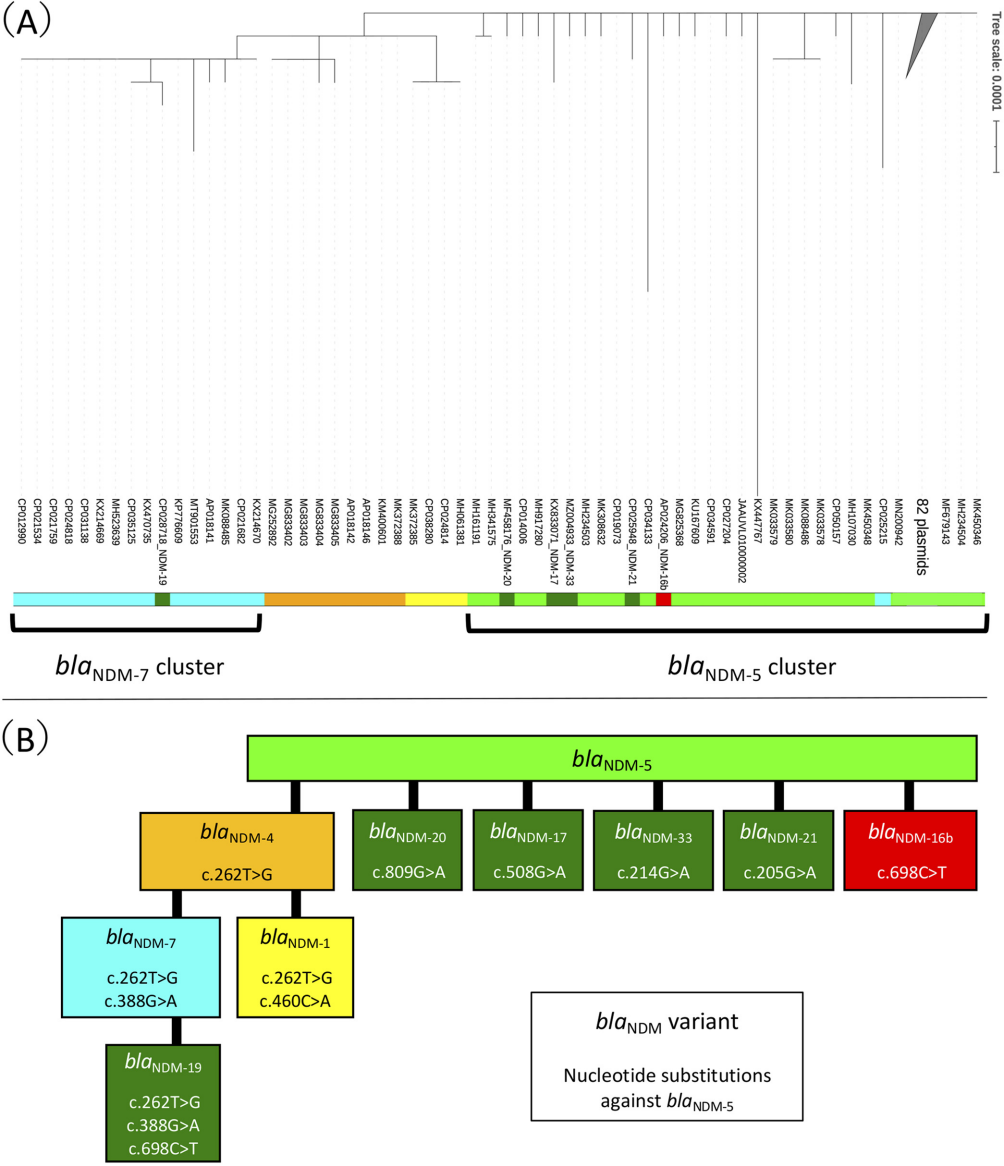
Comparative analysis of *bla*_NDM_-carrying IncX3 plasmids. (A) Maximum-likelihood phylogenetic tree based on shared genes with nucleotide substitutions in the *bla*_NDM_-carrying IncX3 plasmids. The 82 branches (all *bla*_NDM-5_ branches) with high similarity were collapsed using the function of iTOL and were replaced with “82 plasmids.” The color code is as follows: yellow, *bla*_NDM-1_; orange, *bla*_NDM-4_; lime green, *bla*_NDM-5_; cyan, *bla*_NDM-7_; red, *bla*_NDM-16b_; deep green, *bla*_NDM-17,_ *bla*_NDM-19,_ *bla*_NDM-20,_ *bla*_NDM-21_, and *bla*_NDM-33_. The scale distance corresponds to the number of substitutions per site. (B) Flowchart of *bla*_NDM_ nucleotide substitutions. Variant nucleotide substitutions in *bla*_NDM_ compared with *bla*_NDM-5_ (813 bp). Each sequence, excluding *bla*_NDM-33_, is referenced to the National Database of Antibiotic Resistant Organisms (NDARO). The RefSeq ID of each reference is as follows: NG_049326, *bla*_NDM-1_; NG_049336, *bla*_NDM-4_; NG_049337, *bla*_NDM-5_; NG_049339, *bla*_NDM-7_; NG_074726, *bla*_NDM-16b_; NG_052662, *bla*_NDM-17_; NG_055498, *bla*_NDM-19_; NG_057455, *bla*_NDM-20_; NG_055664, *bla*_NDM-21_. The sequence of *bla*_NDM-33_ is referenced to GenBank accession number MZ004933.

We retrieved 142 sequences of *bla*_NDM_-carrying IncX3 plasmids, including pTMTA8571-1 (Table S1). The mean (±standard deviation [SD]) size of these plasmids was 46,923 bp (±3,848 bp). The sequence data revealed the number of plasmid sequences harboring each gene type was as follows: *bla*_NDM-5_ (*n* = 107 sequences); *bla*_NDM-7_ (*n* = 16); *bla*_NDM-4_ (*n* = 9); *bla*_NDM-1_ (*n* = 4); and *bla*_NDM-16b_, *bla*_NDM-17_, *bla*_NDM-19_, *bla*_NDM-20_, *bla*_NDM-21_, and *bla*_NDM-33_ (*n* = 1). According to Roary analysis, IncX3 plasmids carried 34 shared genes (shared genes are defined as genes shared between 99% or more of the plasmid data set), and alignment of the shared genes revealed a sequence of 22,846 bp (Table S2). The phylogenetic tree based on nucleotide substitutions in the shared genes is shown in Fig. 2A. The mean (±SD) for nucleotide substitutions in the shared genes was estimated to be 1.08 (±2.34). The *bla*_NDM_ variants are divided into groups. The *bla*_NDM_ variants, including *bla*_NDM-16b_, *bla*_NDM-17_, *bla*_NDM-20_, *bla*_NDM-21_, and *bla*_NDM-33_, possessed one nucleotide substitution compared with *bla*_NDM-5_ and belonged to the *bla*_NDM-5_ cluster, whereas the *bla*_NDM-19_ variant possessed three nucleotide substitutions compared with *bla*_NDM-5_ (and one nucleotide substitution compared with *bla*_NDM-7_) and belonged to the *bla*_NDM-7_ cluster. Additionally, the phylogenetic tree structure was similar to that of the flowchart of *bla*_NDM_ nucleotide substitutions (Fig. 2B). When a phylogenetic tree was created by excluding *bla*_NDM_ sequences from the shared genes alignment, it was observed that the structure was disrupted and branches were shortened (Fig. S2). Therefore, the IncX3 shared genes phylogenetic tree depended on the inclusion of *bla*_NDM_ gene mutants. In other words, regardless of the *bla*_NDM_ variants, *bla*_NDM_-carrying IncX3 plasmids may have evolved from the same ancestral plasmid, with minimal mutation of the shared genes. It was thereby proposed that the *bla*_NDM-16b_-carrying IncX3 plasmid (pTMTA8571-1) emerged from a *bla*_NDM-5_-carrying IncX3 plasmid.

The IncX3 plasmids analyzed in this study were almost all detected in China (Table S1). These plasmids were detected not only in humans but also in food, animals, and the environment. A few plasmids were detected in Canada, the Czech Republic, Nigeria, Australia, the United Kingdom, Japan, and other parts of the world, except for South America. The organisms harboring the IncX3 plasmid were mainly E. coli and Klebsiella pneumoniae, but other *Enterobacterales* have also been reported (8, 16). Therefore, it is possible to detect *bla*_NDM_-carrying IncX3 plasmids in any host around the world.

Our study had several limitations. First, we analyzed *bla*_NDM_-only-carrying IncX3 plasmids (excluding those coharboring *bla*_NDM_ and other *bla* genes). Therefore, a comparative analysis of the shared genes between all *bla*_NDM_-carrying IncX3 plasmids is yet to be performed. Currently, no established typing method for IncX3 plasmids, such as plasmid multilocus sequence typing (MLST) (https://pubmlst.org/organisms/plasmid-mlst), is available. Previous reports analyzed whole genomes or parts of sequences among a small number of samples, whereas, in this study, we performed a large-scale analysis of the *bla*_NDM_-carrying IncX3 plasmids. To perform a large-scale, high-resolution analysis, we selected shared genes-based comparative analysis of the *bla*_NDM_ only-carrying IncX3 plasmids. If we added the frequently reported *bla*_SHV_- and *bla*_NDM_-carrying IncX3 plasmids (17) to this analysis, the number of shared genes decreased from 34 to 11 (Fig. S3); we were unable to perform a high-resolution analysis. Therefore, we excluded IncX3 plasmids coharboring *bla*_NDM_ and other *bla* genes. Second, the ancestral plasmid of *bla*_NDM_ only-carrying IncX3 plasmids is unknown. The first report of a *bla*_NDM_-carrying IncX3 plasmid was also shown to coharbor the *bla*_NDM_ and *bla*_SHV_ genes (18). It is unclear whether the plasmids in our study emerged from this ancestral plasmid.

In conclusion, to our knowledge, this study is the first to report the molecular characterization of the *bla*_NDM-16b_-carrying IncX3 plasmid. This plasmid is resistant to β-lactam antibiotics and is transferred by conjugation. Additionally, this plasmid may emerge from the widely disseminated *bla*_NDM-5_-carrying IncX3 plasmid by mutation. The *bla*_NDM_-carrying IncX3 plasmids harbored highly similar shared genes and have been reported in many cases worldwide. Because these plasmids may be a hotbed for the emergence of rare or novel variants of *bla*_NDM_, such as in this case, increased attention should be paid to *bla*_NDM_-carrying IncX3 plasmids in the future.

## MATERIALS AND METHODS

### Bacterial strain.

TA8571 strain was isolated from a urine specimen from a hospital inpatient in Tokyo, Japan, in 2018. The isolate was identified as carbapenem-resistant Escherichia coli at the hospital laboratory. The biochemical profile was evaluated with an ID 32E microorganism identification kit (Sysmex bioMérieux Co., Ltd., Tokyo, Japan).

### Antimicrobial susceptibility testing.

Antimicrobial susceptibility testing was performed with the broth dilution method in accordance with the Clinical and Laboratory Standards Institute (CLSI) document M07 guidelines (19). The following antimicrobial agents were used for antimicrobial susceptibility testing: ceftazidime, aztreonam, piperacillin, tazobactam, ciprofloxacin, sulfamethoxazole, and trimethoprim (Tokyo Chemical Industry Co., Ltd., Tokyo, Japan); meropenem, cefotaxime, and gentamicin (Wako Pure Chemical Industries, Ltd., Tokyo, Japan); moxalactam and amikacin (Sigma-Aldrich Inc., St. Louis, MO, USA); imipenem (Combi-Blocks, Inc., San Diego, CA, USA); and cefepime (Toronto Research Chemicals, Toronto, Ontario, Canada). MIC values ranged from 0.12 to 256 μg/mL (trimethoprim/sulfamethoxazole from 0.015/0.3 to 32/608 μg/mL). E. coli ATCC 25922 and Pseudomonas aeruginosa ATCC 27853 were used as the quality control strains for antibiotic susceptibility testing. The results were interpreted in accordance with CLSI guidelines (20).

### Phenotypic and genetic analysis of carbapenem resistance.

Carbapenemase production was confirmed by the mCIM (21). The detection of carbapenemase genes (*bla*_NDM_, *bla*_IMP_, *bla*_VIM-2_, *bla*_KPC_, *bla*_OXA-48_, *bla*_KHM_, *bla*_SMB_, and *bla*_GES_) was performed by PCR with reference to the manual of the National Institute of Infectious Diseases in Japan (https://www.niid.go.jp/niid/images/lab-manual/ResistantBacteria20200604.pdf). Subsequently, the detected *bla* gene was sequenced.

### Bacterial conjugation.

Transfer of the plasmid was confirmed by a filter-mating method with some modifications (22). Briefly, the recipient was a sodium azide-resistant E. coli J53 strain. TA8571 and E. coli J53 were mixed in a 1:1 ratio, and then the mixture was cultured at 37°C for 4 h. The donor, recipient, and transconjugant were selected on LB agar (MP Biomedicals LLC., Solon, OH, USA) supplemented with 100 μg/mL ampicillin (Tokyo Chemical Industry Co., Ltd.) and 100 μg/mL sodium azide (Kanto Chemical Co., Inc., Tokyo, Japan). The transfer frequency was calculated as the colonies of transconjugant per donor colony. The transconjugant was tested for antibiotic susceptibility, and the presence of carbapenemase was confirmed by the mCIM. The detection of carbapenemase genes was performed by PCR and sequencing as described above. PCR amplification of *bla*_CTX-M-14_ (*bla*_CTX-M-9-group_) was performed to rule out the possibility of contamination of the donor strain TA8571 (23).

### S1 pulsed-field gel electrophoresis.

S1-PFGE was performed in accordance with the method of Barton et al. (24) with some modifications. Briefly, DNA plugs digested with S1 nuclease (TaKaRa Bio Inc., Shiga, Japan) were electrophoresed on a CHEF Mapper XA PFGE system (Bio-Rad Laboratories, Inc., Hercules, CA, USA) with autoalgorithms for 5 to 500 kbp. A lambda ladder (Promega Co., Fitchburg, WI, USA) was used as the size marker.

### Whole-genome sequencing analysis.

A short-read library was prepared using the Nextera XT DNA library prep kit (Illumina, Inc., San Diego, CA, USA) from the DNA extracted using a QIAamp DNA minikit (Qiagen GmbH, Hilden, Germany) and was sequenced with a MiSeq instrument using a MiSeq reagent v3 kit (600 cycles; Illumina). A long-read library was prepared using a ligation sequencing kit and a native barcoding expansion 1-12 kit and sequenced with a MinION sequencer (Oxford Nanopore Technologies Ltd., Oxford, UK). Hybrid assembly of both the short and long reads was performed with Unicycler v0.4.9b (25). Genes were predicted and annotated using the DNA Data Bank of Japan (DDBJ) Fast Annotation and Submission Tool (DFAST) under default parameters (26). In the chromosome analysis, species identification was performed by average nucleotide identity (ANI) analysis for an E. coli type strain ATCC 11775 (27), and multilocus sequence typing (MLST) was performed by MLST 2.0 (28). Chromosomal point mutations in the quinolone resistance-determining regions and antibiotic resistance genes were determined by ResFinder 3.4 (29). In the plasmid analysis, acquired antibiotic resistance genes were determined by ResFinder, and plasmid incompatibility replicon typing was performed with PlasmidFinder 2.0 (30). Genome representation was performed using the CGview server (http://cgview.ca/).

### Comparative analysis of *bla*_NDM_-carrying IncX3 plasmids.

Complete IncX3 plasmid sequences were retrieved from the GenBank database and previous reports using a keyword search for the words “complete sequence” and “IncX3 plasmid.” For the retrieved sequences, ResFinder and PlasmidFinder searches were performed as described above, and only selected complete sequences of *bla*_NDM_-carrying IncX3 plasmids (except for those coharboring *bla*_NDM_ and other *bla* genes). Comparative analysis of shared genes of *bla*_NDM_-carrying IncX3 plasmids was performed with Roary v3.13.0 using default parameters (31). GFF files used in Roary were output from DFAST using default parameters (26). A maximum-likelihood phylogenetic tree was constructed with FastTree v2.1.10 under the general time-reversible model with a categorical model of rate heterogeneity (GTR-CAT), based on the Roary method of alignment (32). The phylogenetic tree can be viewed using iTOL (https://itol.embl.de).

### Ethical approval.

According to the bylaws of the Tokyo Metropolitan Institute of Public Health, the study was approved by the ethical review committee of the Tokyo Metropolitan Institute of Public Health (approval no. 30-782).

### Data availability.

The DDBJ accession numbers for sequences obtained in this study are as follows: AP024205 (TA8571 chromosome), AP024206 (pTMTA8571-1), AP024207 (pTMTA8571-2), AP024208 (pTMTA8571-3), and AP024209 (pTMTA8571-4).
