# Supplementary material for: Molecular Characterization of blaNDM-Carrying IncX3 Plasmids: blaNDM-16b Likely Emerged from a Mutation of blaNDM-5 on IncX3 Plasmid
Source: Microbiol Spectr. 2022 Jul 13;10(4):e01449-22. doi: 10.1128/spectrum.01449-22 (PMC9430178; doi:10.1128/spectrum.01449-22)
Supplement: Supplemental file 1 — Supplemental material. Download spectrum.01449-22-s0002.pdf, PDF file, 0.9 MB [file spectrum.01449-22-s0002.pdf]

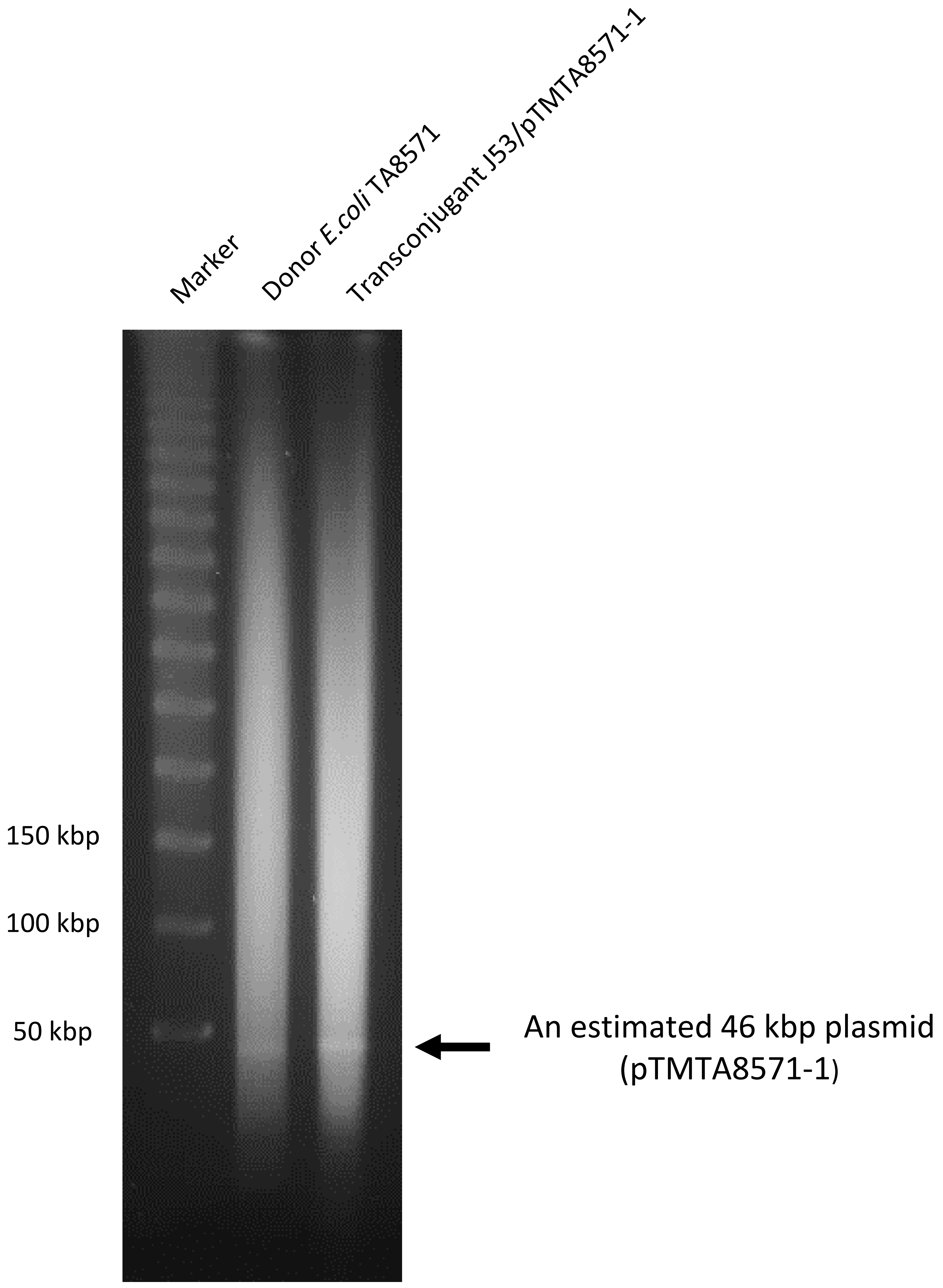

Figure S1. An image of S1-PFGE pattern observed in *E.coli* TA8571 and transconjugant J53/pTMTA8571-1. A lambda ladder (Promega Co., Fitchburg, WI, USA) was used as the size marker.

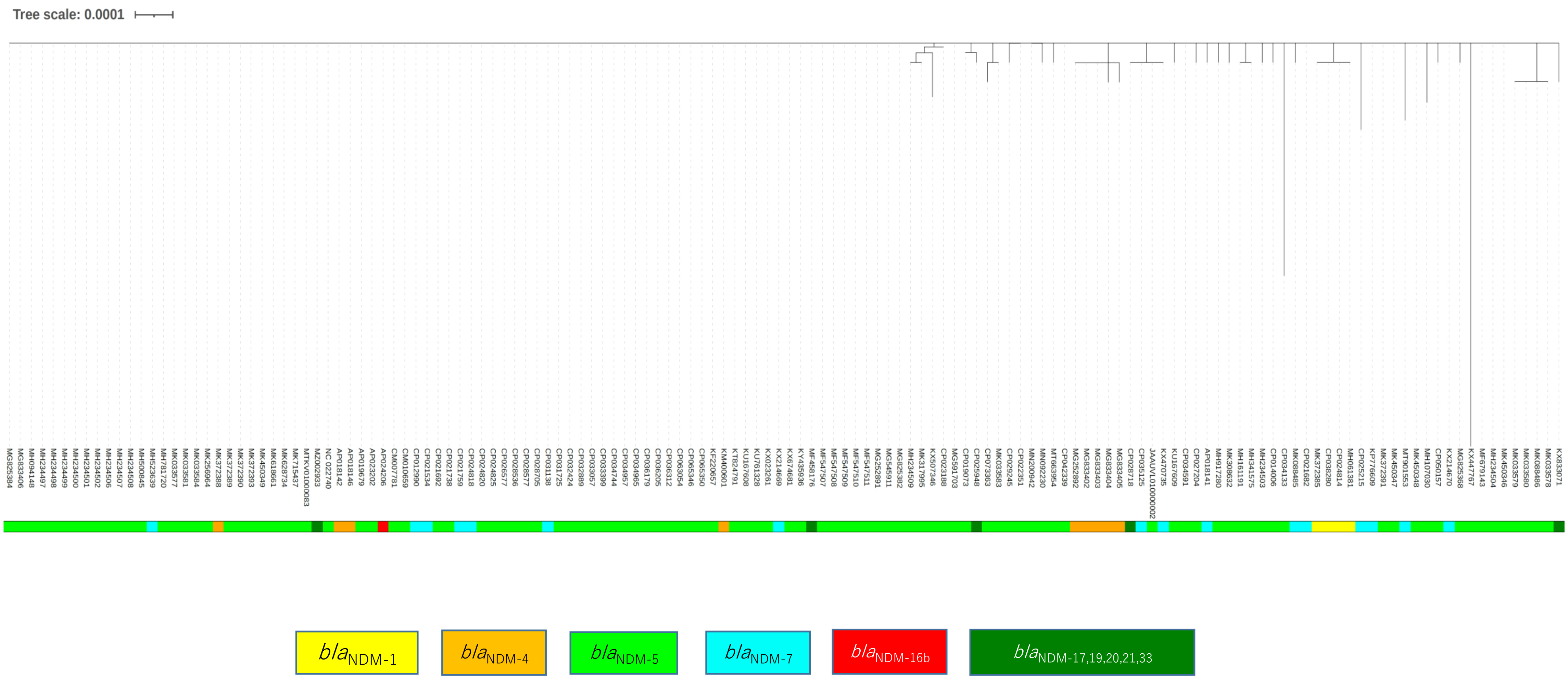

Figure S2. Phylogenetic tree based on shared-genes alignment excluding *bla*$_{NDM}$ sequences.

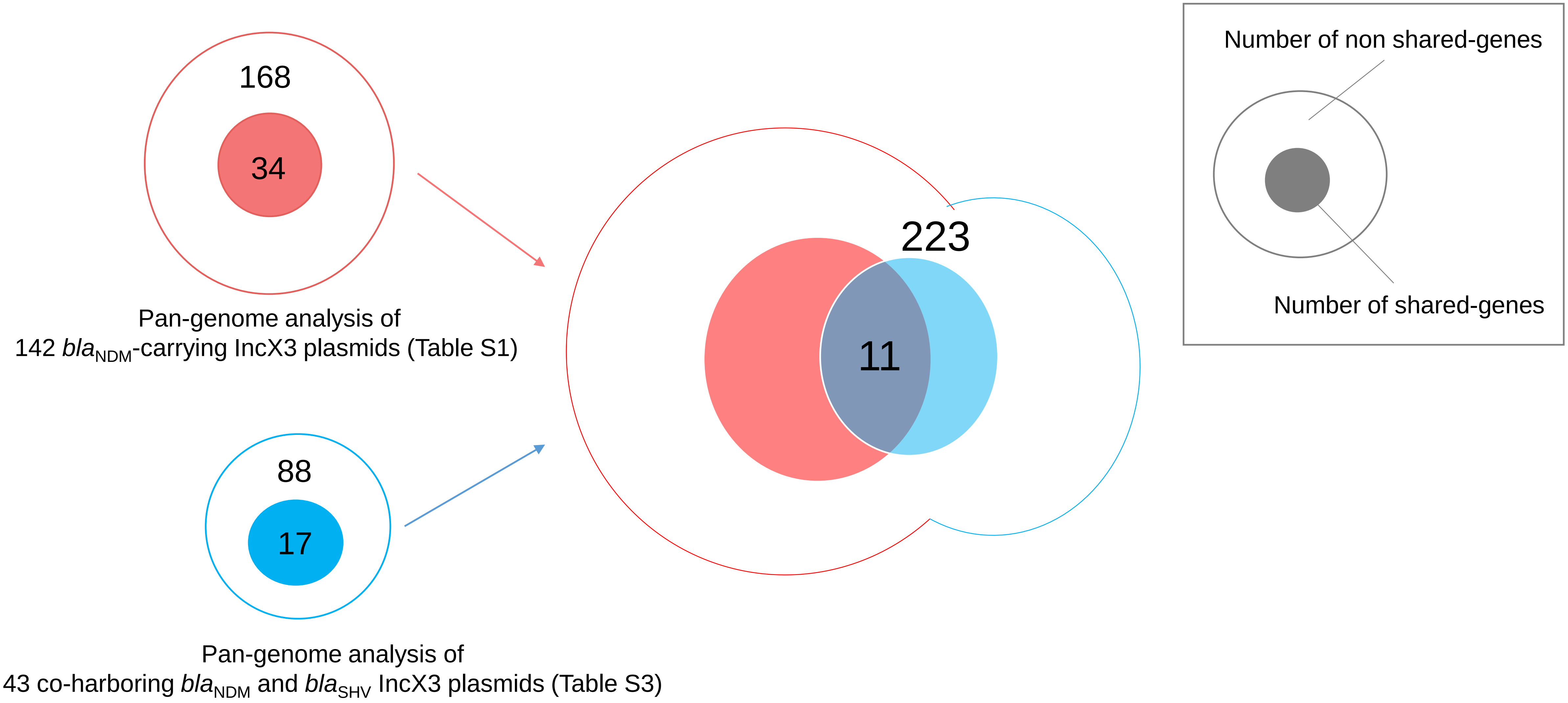

Figure S3. Pan-genome analysis of *bla*$_{NDM}$-carrying IncX3 plasmids including co-harboring *bla*$_{NDM}$ and *bla*$_{SHV}$ plasmids using by Roary.
